# Vascular, physical fitness, lifestyle, and body composition characteristics in middle-aged and older diver fishermen: association between shear rate and lower-limb physical fitness

**DOI:** 10.3389/fphys.2026.1739696

**Published:** 2026-03-17

**Authors:** Alex Véliz, Raquel Pereira Berríos, Anita Dörner Paris, David C. Andrade, Cristian Álvarez

**Affiliations:** 1 Departamento de Ciencias Sociales, Universidad de Los Lagos, Osorno, Chile; 2 Departamento de Ciencias de La Actividad Física, Universidad de Los Lagos, Puerto Montt, Chile; 3 Departamento de Salud, Universidad de Los Lagos, Osorno, Chile; 4 Exercise Applied Physiology Laboratory, Centro de Investigación en Fisiología y Medicina de Altura (FIMEDALT), Departamento Biomédico, Facultad de Ciencias de la Salud, Universidad de Antofagasta, Antofagasta, Chile; 5 Exercise and Rehabilitation Sciences Institute, School of Physical Therapy, Faculty of Rehabilitation Sciences, Universidad Andres Bello, Santiago, Chile

**Keywords:** body composition, carotid intima–media thickness, dual-X-ray absorptiometry, handgrip strength, peak systolic velocity, pulse wave velocity

## Abstract

**Objectives:**

First, to describe the vascular, physical fitness, lifestyle, and body composition characteristics of middle-aged and older adult diver fishermen. Second, to associate vascular outcomes with physical fitness (upper and lower limbs).

**Methods:**

A descriptive pilot study was performed in middle-aged [MA-DF, *n* = 11, body mass index (BMI) 29.9 ± 4.9, mean arterial pressure (MAP) 103.9 ± 6.2 mmHg] and older (OA-DF, *n* = 11, BMI 28.5 ± 2.7, MAP 111.8 ± 9.6 mmHg) adult diver fishermen. In each group, brachial (BA) and common carotid artery (CCA) diameter (*D*_BA_; *D*_CCA_), peak systolic (PSV_BA_; PSV_CCA_), end-diastolic velocity (EDV_BA_; EDV_CCA_), shear rate (SR_BA_; SR_CCA_), resistance index (RI_BA_; RI_CCA_), pulsatility index (PI_BA_; PI_CCA_), Reynolds number (Re_BA_; Re_CCA_), handgrip strength right (HGS_RA_), left (HGS_LA_), and average (HGS_AV_) and lower-limb fitness (Ruffier test) were the main outcomes, while other types of information, including vascular ankle-brachial index, pulse wave velocity, carotid intima average and maximum, augmentation index, body composition (segmental and total parameters by dual-X-ray absorptiometry), and lifestyle, were secondary outcomes.

**Results:**

There were no vascular, body composition, or lifestyle differences between groups. The MA-DF group showed superior upper- (HGS_RA_ 48.1 ± 6.2 kg vs. 39.8 ± 6.4 kg; HGS_LA_ 46.7 ± 5.9 kg vs. 39.5 ± 6.3 kg, both *P* < 0.05) and lower-limb fitness (Ruffier test 23.2 ± 5.3 repetitions vs. 15.5 ± 2.4 repetitions, *p* = 0.0006) vs. the OA-DF group. Significant associations were found between SR_BA_ and the Ruffier test (*p* = 0.003) and between SR_CCA_ and the Ruffier test (*p* = 0.042).

**Conclusion:**

Despite similar vascular, lifestyle, and body composition profiles, middle-aged and older diver fishermen displayed marked differences in upper- and lower-limb physical fitness. Importantly, lower-limb physical fitness, as assessed by the Ruffier test, emerged as a robust correlation of vascular shear rate (SR) in both the BA and CCA, highlighting its potential relevance to peripheral and central vascular function.

## 1 Introduction

Diving involves a large population, with approximately nine million divers in the United States (Taylor et al., 2003). Excluding people who dive for tourism, scientific, or recreational aims, diving related to the fishing industry (i.e., aquiculture, salmon, and mollusk harvesting) represents an important occupational activity for young and older adults on the American coasts of the Pacific and Atlantic oceans. In Chile alone, for example, approximately 3,500–4,000 people dive commercially (Souto Cavalli et al., 2023; SUSESO, 2020). Diver fishermen (DF), technically known as “basic shellfish divers” by the Chilean Navy Force authority, show specific physiological adaptations and maladaptations in terms of vascular and body composition health (Armada De Chile, 2014).

Diving work is conducted in a “reduced gravitational environment” that is slightly similar to the zero gravity of space (Turner, 2000), and thus divers with more years of experience could show a reduced bone mineral content (Véliz et al., 2025). Although DF dedicated to mollusk harvesting are frequently doing upper limb movements in 20–36 m of depth, and these actions could not represent a major cardiovascular risk (i.e., under the correct application of the diving decompression rules) (Armada De Chile, 2014), this diving specialty offers a low load on the human skeleton and on lower-limb muscles that could implies potential bone mineral content (BMC) consequences at more adult ages in the long term (Hwang et al., 2006). The diving exposes the body to increased pressure and inert gas saturation, producing unique physiological effects and potential vascular disorders (Bove, 2014). The pressure changes can cause ear, sinus, or lung injuries due to gas volume shifts during ascent/descent, and the supersaturated inert gases may form nitrogen bubbles, leading to decompression disease (Bove, 2014). Other vascular consequences from the hyperbaric environment include endothelial dysfunction, which alters the nitric oxide production, and vasodilation, arterial stiffness, and oxidative stress that alters the endothelial cells (Obad et al., 2010). Conversely, muscular activity may counteract these effects. Muscular movement in the human skeleton, through maintaining a healthy lifestyle, such as adhering to international physical activity guidelines of different intensities and modalities (WHO, 2021), could contribute to muscle mass and strength maintenance of the upper and lower limbs and promote more osteogenic stimulus (Bevier et al., 1989; Li and Wang, 2025).

A recent governmental study conducted by the Chilean Superintendence of Social Security (SUSESO) showed a major prevalence of musculoskeletal disorders, hypertension, working memory impairment, and osteonecrosis in those DF more exposed to hyperbaric conditions (SUSESO, 2020). A more recent report adds that 36% of the DF industry reports a smoking habit and high prevalence of overweight and obesity (∼86.7%), which increases their cardiovascular risk (SUSESO, 2020). As aging is associated with progressive structural and functional deterioration of the vascular system (i.e., biological aging), some physical fitness factors (i.e., muscle strength, functional capacity and cardiorespiratory fitness) and lifestyle patterns (i.e., physical activity patterns and sedentary behavior) could worsen the vascular health and thus accelerate the occurrence of CVD when they are poorly addressed (Seth et al., 2025). Importantly, maintaining upper and lower limb physical fitness, such as muscular strength, functional capacity, or cardiorespiratory fitness, may mitigate some of the deleterious vascular effects of chronological aging (Subramanian et al., 2025). In this line, and with wide knowledge about the deleterious effects of environmental and work contexts in which employees spend much sedentary time, some strategies such as “exercise snacks” have been proposed to avoid early CVD in office workers (Lazić et al., 2025).

The DF of the Chilean coast begin this activity early in life and maintain active occupational work into older ages (i.e., >60 years old). However, there is little information regarding the peripheral and central vascular characteristics, physical fitness, or body composition, particularly in older adult divers. In this regard, handgrip strength (HGS) is a widely recognized marker of upper limb muscle strength, where higher levels show better cardiovascular health. In comparison, lower HGS levels are typically associated with a higher prevalence of CVD in Americans aged 20–60 years (Li and Wang, 2025). On the other hand, recent studies have highlighted the relevance of using simple field-based or clinical tests due to their potential to estimate physical fitness parameters [i.e., cardiorespiratory fitness (CRF), muscle strength fitness, or functional capacity] and thereby help prevent CVD (Vakulenko et al., 2024; Vakulenko and Vakulenko, 2024; González-García and McCarthy, 2022). For lower-limb physical fitness, the Ruffier test was previously associated with CRF and cardiovascular risk (Alahmari et al., 2020), and it is useful for estimating CRF in divers (Sartor et al., 2016). Similarly, the peak oxygen consumption (VO2_peak_), a surrogate for maximal oxygen uptake and frequently used in clinical testing (Zamodics et al., 2025), is a strong predictor of CVD and mortality (Weeldreyer et al., 2025). The Ruffier test has shown significant correlation with femoral artery blood flow recovery in trained athletes and may be useful for monitoring physiological recovery after physical efforts using Doppler ultrasound (Piquet et al., 2000). Previous studies in divers have also reported that they exhibit greater carotid intima–media thickness (cIMT), and lower peak systolic and end-diastolic velocity values [i.e., resulting in different shear rate (SR)] compared with healthy adult controls (Dormanesh et al., 2016). However, it is also well documented that increasing age is associated with a greater increase in arterial stiffness determined by the pulse wave velocity vascular outcome (PWV). For example, hypertensive populations are known to show elevated PWV values (i.e., ≥10 m·s^−1^) that denote elevated CVD risk in adults. At the same time, lifestyle interventions such as exercise can also improve these outcomes (i.e., decrease PWV) (Álvarez et al., 2024). Thus, if DF of different ages present differences in physical fitness and traditional vascular parameters such as cIMT and PWV, it would similarly be reasonable to expect changes in blood flow variables such as shear rate (SR) and the Reynolds number to estimate “laminar” or “retrograde” blood fluxes (Gomez et al., 2022; Tremblay et al., 2019). Evidence is scarce regarding physical fitness and blood flow characteristics in the BA or CCA arteries of adult men exposed to pressure-changing environments, such as DF. The relationship of peripheral and central vascular characteristics in DF with the physical fitness of the upper and lower limbs is less clear, with the aim of detecting their vascular risk and preventing CVD. Assessing these outcomes among active DF of different age groups may elucidate modifiable factors that contribute to healthier vascular or musculoskeletal aging. Therefore, the main aim of this study was to describe the vascular, physical fitness, lifestyle, and body composition characteristics of middle-aged and older adult diver fishermen. The second aim was to associate vascular outcomes with physical fitness (upper and lower limbs). We hypothesize that upper- and lower-limb physical fitness can be associated with peripheral or central vascular parameters of function (i.e., SR_BA_ or SR_CCA_) or structure [i.e., diameters of BA (*D*_BA_) or CCA (*D*_CCA_)] of DF.

## 2 Materials and methods

### 2.1 Study participants

A descriptive pilot study was conducted from June 2024 to April 2025 in adult DF affiliated with social fishermen’s and mollusk harvesting organizations from the coastal cities of Puerto Montt, Calbuco, Maullín, and Carelmapu in southern Chile. Participants were recruited through an open call issued directly to the leaders of each divers’ organization. Prior to enrollment, the study aims, methodological procedures, and the scope of the assessments and counseling services were clearly presented to each organization. The inclusion criteria were as follows: *i*) age ≥18 years (legal adulthood under Chilean law); *ii*) active occupational diver; *iii*) membership in a recognized divers’ social or professional organization; and *iv*) availability to attend all study assessments. The exclusion criteria included *i*) any contraindication to undergoing an iDXA assessment involving X-ray exposure; *ii*) the presence of electronic medical devices, such as pacemakers or insulin pumps, that could interfere with iDXA evaluation; and *iii*) any condition that prevented the completion of non-invasive ultrasound assessments.

**FIGURE 1:**
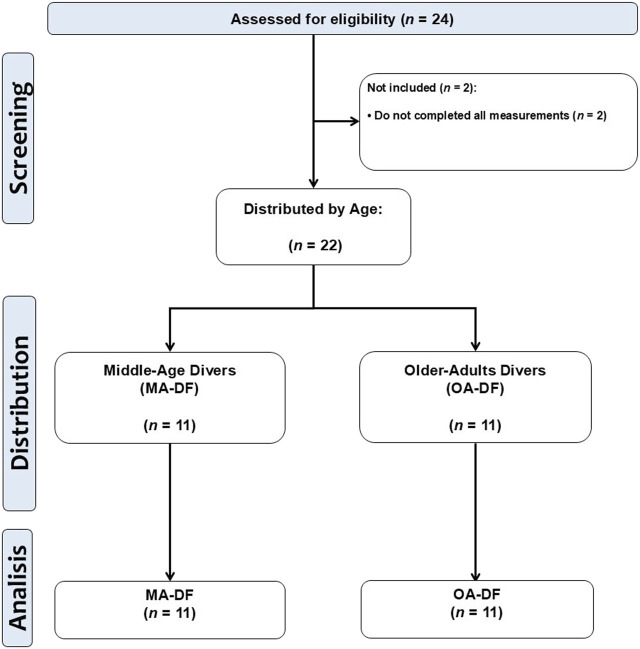
CONSORT study design flow chart.

The research protocol received ethical approval from the Scientific Ethical Committee of the Universidad Mayor under approval number N° 0492 of 2024. All individuals who took part provided written informed consent, which included detailed information on the evaluation procedures, confidentiality, data protection measures, anonymity assurances, and a description of any potential risks. The sample size for this pilot descriptive study was decided based on prior data from a similar cohort of adult participants, using the body mass index (BMI) as a reference outcome. A standard deviation (SD) of ≤5.0 kg/m^2^ was used to represent expected interindividual variability in BMI within each group, consistent with previously reported values in comparable populations (Perovic et al., 2017). Thus, considering the exploratory nature of the study, a total of (*n* = 22) participants, divided into two groups (*n* = 11 per group), were selected to detect preliminary trends and inform future sample size estimations. A post-hoc power analysis conducted with G*Power software (version 3.1.9.6, Franz Faul, Universität Kiel, Kiel, Germany) revealed that with this SD and a moderate effect size (Cohen’s *d* = 0.5), the current sample provides an estimated statistical power of approximately 80% at a significance level of α = 0.05 (two-tailed). This sample is considered proper for pilot-level research and feasibility assessment of the study protocol. The final sample included groups of middle-aged diver fishermen (MA-DF, *n* = 11) and older adult diver fishermen (OA-DF, *n* = 11). The CONSORT study design can be seen in (Figure 1).

### 2.2 Pulse wave velocity and co-variables

Arterial stiffness was measured by the pulse wave velocity (PWV) outcome. This outcome was measured in the BA using oscillometric pressure traces from the BA in the upper left arm in 90° abduction (measured in m·s^−1^) with an automatic digital device after a 20-min rest in a supine position (Arteriograph, TensioMed™, Hungary). Data analysis was conducted with the Arteriograph software v.1.9.9.2. This equipment operates similarly to a blood pressure assessment using a validated algorithm (Ring et al., 2014), and a PDF report was extracted as a result. The ankle-brachial index (ABI) and the augmentation index of the BA (AIx_BA_) were also extracted and registered. PWV values exceeding 10 m·s^−1^ indicate elevated arterial stiffness, correlating with increased CVD risk (Mancia et al., 2013).

### 2.3 Brachial and common carotid artery diameter and flow characteristics

First, the BA artery diameter (*D*_BA_) was continuously monitored using high-resolution ultrasound imaging (GE Medical Systems, Model LOGIQ-E PRO, Milwaukee, United States), after 20 min of rest, similar to previous studies (Thijssen et al., 2019). Shear rate of the BA (SR_BA_) was calculated using peak systolic velocity BA (PSV_BA_) and *D*_BA_ diameter using the formula SR_BA_ = 4 · PSV_BA_/*D*_BA_, providing insights into endothelial shear stress during rest. The pulsatility index of BA (PI_BA_) was calculated as PI_BA_ = PSV_BA_ − EDV_BA_/V_av_, where: PSV_BA_ = peak systolic velocity, EDV_BA_ = end-diastolic velocity, and V_av_ = average velocity, calculated as V_av_ = PSV_BA_ + 2 · EDV_BA_/3.

The resistivity index of BA (RI_BA_) was derived from Doppler waveform analysis, reflecting vascular resistance and pulsatile flow dynamics (Natale et al., 2014), and calculated as RI_BA_ = PSV_BA_ − EDV_BA_/PSV_BA_. Additionally, the Reynolds number of BA (Re_BA_) was estimated to assess the nature of blood flow (anterograde or retrograde), considering the following formula: Re_BA_ = *ρ* · PSV_BA_ · *D*_BA_/*μ*, where *ρ =* blood density (1.06 g/cm^3^), PSV_BA_ = peak systolic velocity, *D*_BA_ = brachial artery diameter, and *μ =* blood viscosity 0.035 g/(cm·s). These variables were selected to comprehensively evaluate *D*_BA_ and flow characteristics in the left BA under resting conditions. The same vascular outcomes were measured in the central CCA in terms of diameter (*D*_CCA_) and fluxes (PSV_CCA_; EDV_CCA_, SR_CCA_, RI_CCA_, PI_CCA_, Re_CCA_).

The carotid intima–media thickness average (cIMT_av_) and maximum (cIMT_max:_, defined as the maximum thickness within the cIMT_av_ measurement) were obtained from the CCA by an ultrasound imaging 7–12 MHz linear-array transducer (GE Medical Systems, Model LOGIQ-E PRO, Milwaukee, United States). After carotid bulb identification, an image was obtained in “B mode” in longitudinal orientation of the right CCA by an automatic ultrasound function that detects both cIMT_av_ and cIMT_max_ outcomes. The scan was focused 1 cm from the carotid bifurcation on the far wall. The ultrasound software recorded the image, and later, it was analyzed offline. All measurements were recorded at the end-diastolic stage (Coll and Feinstein, 2008). Given that the cIMT_av_ >0.9 mm has been used as a previous cut-off point to denote high cardiovascular risk, we used this value in our cIMT_max_ outcome, following the European Society of Hypertension and European Society of Cardiology recommendations (Mancia et al., 2013).

### 2.4 Blood pressure

Blood pressure was measured on three attempts with rest intervals of at least 1 min between measurements, using a digital cuff instrument positioned on the arm Omron™ (Model HEM-7142, United States). Blood pressure was categorized according to the criteria of the European Society of Cardiology into hypertension [systolic blood pressure (SBP) ≥140 or diastolic blood pressure (DBP) ≥90 mmHg], normal-high blood pressure (SBP 130–139 mmHg or DBP 85–89 mmHg), and normotensive (SBP ≤129 mmHg or DBP ≤84 mmHg) (Marx et al., 2023).

### 2.5 Physical fitness of the upper limb and lower limb

For HGS, measurements of both hands were used in three attempts in a seated position, and we used the HGS of the right (HGS_RA_: HGS right arm) and left arm (HGS_LA_: HGS left arm) as the upper limb physical fitness. Additionally, the average of HGS_RA_ plus HGS_LA_ [i.e., handgrip strength average (HGS_AV_)] was used as the upper limb marker in the associative analyses. These measurements were developed by using a digital handheld dynamometer (Jamar®, Plus+, Sammons Preston, Patterson Medical, Illinois, United States) following previous studies in Latin American populations (Santos et al., 2025).

For lower-limb physical fitness, we used the Ruffier–Dickson test to calculate the Ruffier index (RI) (Joussellin, 2007). The participants were instructed as follows: 1) the heart rate at rest was in (beats per minute) and registered as HR1, then the person stood up and 2) executed a squat exercise at a steady pace with the thighs straight at 90° of knee flexion, raising the arms forward while flexing for 45 s. Immediately after performing the 45-s squat exercise, 3) the heart rate was taken again and recorded as HR2, followed by a 1-min rest in a seated position, and 4) finally, the heart rate was recorded again as HR3. The results were then interpreted using the following formula: Ruffier index = [(HR1 + HR2 + HR3) − 200]/10, where the squat repetitions and heart rate values could be useful for estimating the CRF (data not shown).

### 2.6 Lifestyle patterns

The physical activity (PA) levels of the participants were determined with the Global Physical Activity Questionnaire (GPAQ v2) (WHO, 2009). From here, the min/week of vigorous (PA_VI_), moderate (PA_MI_), and light PA (PA_LI_) was determined and registered by each participant. The sedentary time per week was determined by self-reporting time spent on activities involving sitting or reclining during leisure time. To determine the smoking habit, the smoking surveillance instrument proposed by the Pan-American Health Organization was used. This instrument identified current smokers (daily and occasionally) and ex-smokers, allowing the assessment of the number of cigarettes smoked and the persistence of the habit, but in this study, we registered the data as non-smoker or smoker (Guatibonza-García et al., 2025).

### 2.7 Anthropometry (secondary outcomes)

Weight was measured with the BIA equipment InBody120™ scale (tetrapolar 8-point tactile electrode system, model BPM040S12F07, BioSpace, Inc., Seoul, Korea) with 0.1 kg precision following previous studies (Marfell-Jones et al., 2006). Height was measured with a SECA™ 213-Topmedic portable stadiometer (Germany). BMI was calculated using weight and height squared.

### 2.8 Body composition by dual-X-ray absorptiometry (secondary outcomes)

**FIGURE 2:**
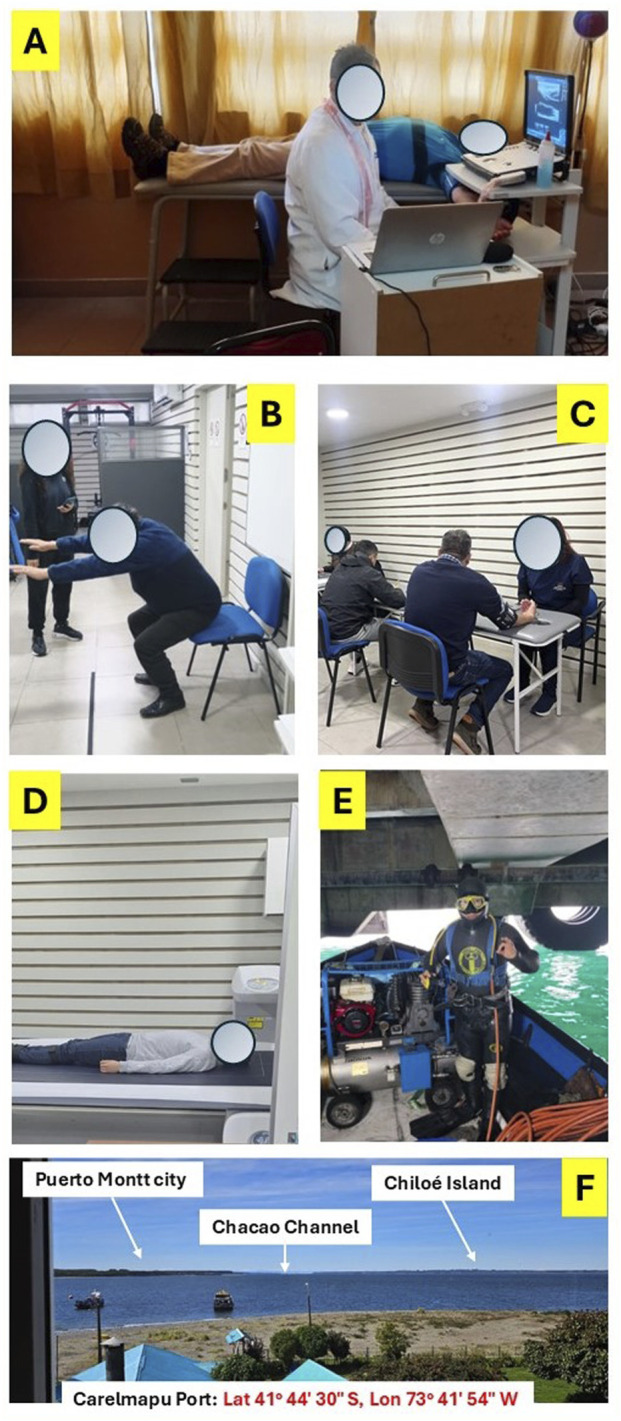
Vascular measurement **(A)**, physical fitness Ruffier test **(B)**, lifestyle patterns [Global Physical Activity Questionnaire (GPAG)] **(C)**, body composition iDXA measurement **(D)**, equipment characteristics **(E)**, and georeferenced geographic area of the Carelmapu Port (Southern Chile), as the city of the diver fishing participants **(F)**.

The participants visited the Universidad de Los Lagos Laboratory (Puerto Montt city, Chile) for the iDXA body composition evaluation from Monday to Friday between 9 a.m. and 1 p.m. to perform these procedures. Prior to the iDXA measurement (Healthcare General Electric Company, Encore 18 Software, United States), a preliminary interview was conducted to rule out the use of electronic devices such as pacemakers and insulin pumps, among others, that could interfere with the operation of the equipment or could affect the health of the participant. For the iDXA measurement, each subject was placed in a supine position on the equipment, wearing light clothing, without shoes or metal objects. All iDXA measurements were applied segmentally [i.e., body fat % arms (BF_Arms_), left arm (BF_LA_), right arm (BF_RA_), legs (BF_Legs_), right leg (BF_RL_), left leg (BF_LL_), and trunk (BF_Trunk_); fat-free mass g arms (FFM_Arms_), left arm (FFM_LA_), right arm (FFM_RA_), legs (FFM_Legs_), right leg (FFM_RL_), left leg (FFM_LL_), and trunk (FFM_Trunk_); and bone mineral content of arms (BMC_Arms_), left arm (BMC_LA_), right arm (BMC_RA_), legs (BMC_Legs_), right leg (BMC_RL_), left leg (BMC_LL_), and trunk (BMC_Trunk_)]; and in total parameters (i.e., total body fat % (Total BF), total fat-free mass (Total FFM), and total bone mineral content (Total BMC)). The evaluation process lasted approximately 10 min. Parts of the vascular, physical fitness, body composition, and equipment characteristics of the DF can be seen in Figure 2.

### 2.9 Statistical analysis

Data are shown as mean and (±) standard deviation. The Shapiro–Wilk test was applied to test the normal distribution of the main and secondary outcomes. For outcomes with normal distribution, the differences between groups were evaluated by paired or unpaired *t*-test at a *P* < 0.05 alpha error level. Additionally, Cohen’s *d* effect size was described. The chi-square test was applied at a *P* < 0.05 alpha level to assess frequencies of blood pressure categories. The Wilcoxon test was applied to those outcomes with no normal distribution. Multivariable regression (i.e., adjusted by age and MAP outcomes) was applied to test the association between vascular outcomes of the BA: *D*_BA_, SR_BA_, CCA, *D*_CCA_, and SR_CCA_, with upper (HGS_AV_) and lower-limb physical fitness (Ruffier test repetitions). The analyses were performed using GraphPad Prism version 8.0 statistical software (Chicago, Illinois, United States).

## 3 Results

### 3.1 General and brachial artery characteristics

Comparing the MA-DF vs. OA-DF groups, there were significant differences in characteristics of age (48.0 ± 8.5 years vs. 66.0 ± 5.9 years, *p* = 0.0006), systolic (138.2 ± 6.0 mmHg vs. 150.1 ± 16.3 mmHg, *p* = 0.043), and mean arterial pressure (103.9 ± 6.2 mmHg vs. 111.8 ± 9.6 mmHg, *p* = 0.039) (Table 1). No other differences were detected in the general characteristics of the sample (Table 1).

**TABLE 1:** Anthropometric and body composition characteristics of participants.

Outcome	MA-DF	OA-DF	*p-*value, *d*
Age (y)	48.0 ± 8.5	66.0 ± 5.9	***p* = 0.0006, 0.70**
Height (cm)	169.7 ± 5.3	171.8 ± 5.4	*p* = 0.445, 0.03
Weight (kg)	86.0 ± 12.6	84.4 ± 10.1	*p* = 0.775, 0.005
Body mass index (kg/m^2^)	29.9 ± 4.9	28.5 ± 2.7	*p* = 0.464, 0.03
*Blood pressure*			
Systolic BP (mmHg)	138.2 ± 6.0	150.1 ± 16.3	***p =* 0.043, 0.19**
Diastolic BP (mmHg)	87.0 ± 6.7	92.6 ± 7.6	*p =* 0.082, 0.14
Mean arterial pressure (mmHg)	103.9 ± 6.2	111.8 ± 9.6	***p* = 0.039, 0.20**
*Blood pressure categorization*			
Normal (*n* =)/%	2 (18.1)	0 (0)	*p* = 0.123^#^
High blood pressure (*n* =)/%	6 (54.5)	4 (36.3)	
Hypertensive (*n* =)/%	3 (27.2)	7 (63.6)	

Data are shown as mean ± SD. Groups are described as middle-aged diver fishermen (MA-DF) and older adult diver fishermen (OA-DF).

*d*, Cohen’s *d* effect size. # denotes chi-square test applied.

**FIGURE 3:**
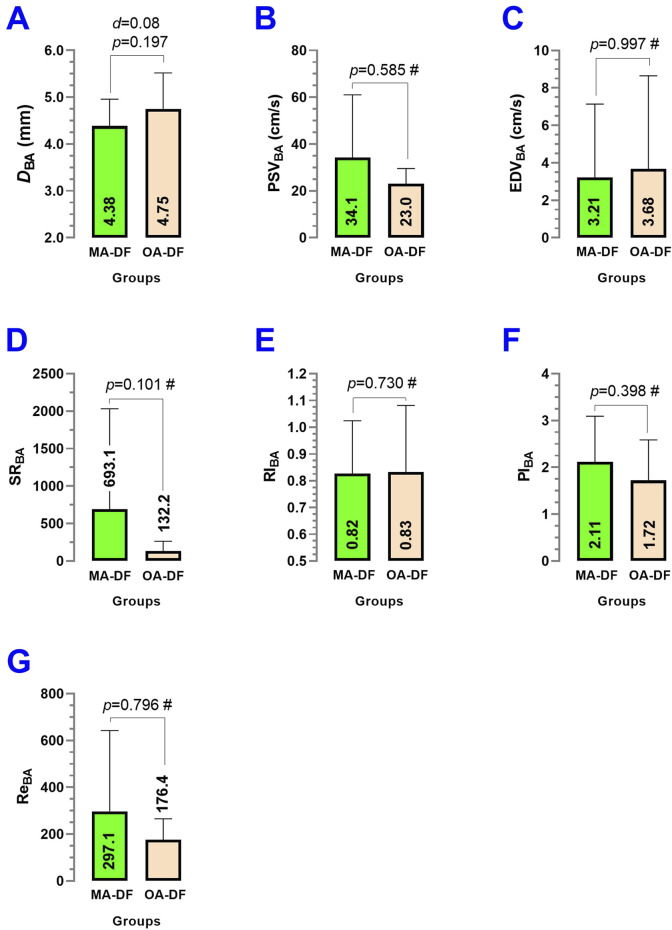
Brachial artery diameter (*D*_BA_) **(A)**, peak systolic velocity (PSV_BA_) **(B)**, end-diastolic velocity (EDV_BA_) **(C)**, shear rate (SR_BA_) **(D)**, resistance index (RI_BA_) **(E)**, pulsatility index (PI_BA_) **(F)**, and Reynolds number (Re_BA_) **(G)** in diver fishermen of the southern Chilean Coast dedicated to mollusk harvesting. Groups are described as middle-aged diver fishermen (MA-DF) and older adult diver fishermen (OA-DF). (#) Analyzed by the unpaired *t*-test at *p* < 0.05. (*d*) Cohen’s *d* effect size at *p* < 0.05.

In the BA, comparing MA-DF vs. OA-DF, the *D*_BA_ (*p* = 0.197) (Figure 3A), PSV_BA_ (*p* = 0.585) (Figure 3B), EDV_BA_ (*p* = 0.997) (Figure 3C), SR_BA_ (*p* = 0.101) (Figure 3D), RI_BA_ (*p* = 0.730) (Figure 3E), PI_BA_ (*p* = 0.398) (Figure 3F), and Re_BA_ (*p* = 0.796) (Figure 3G) showed no significant differences between groups.

### 3.2 Common carotid artery characteristics

**FIGURE 4:**
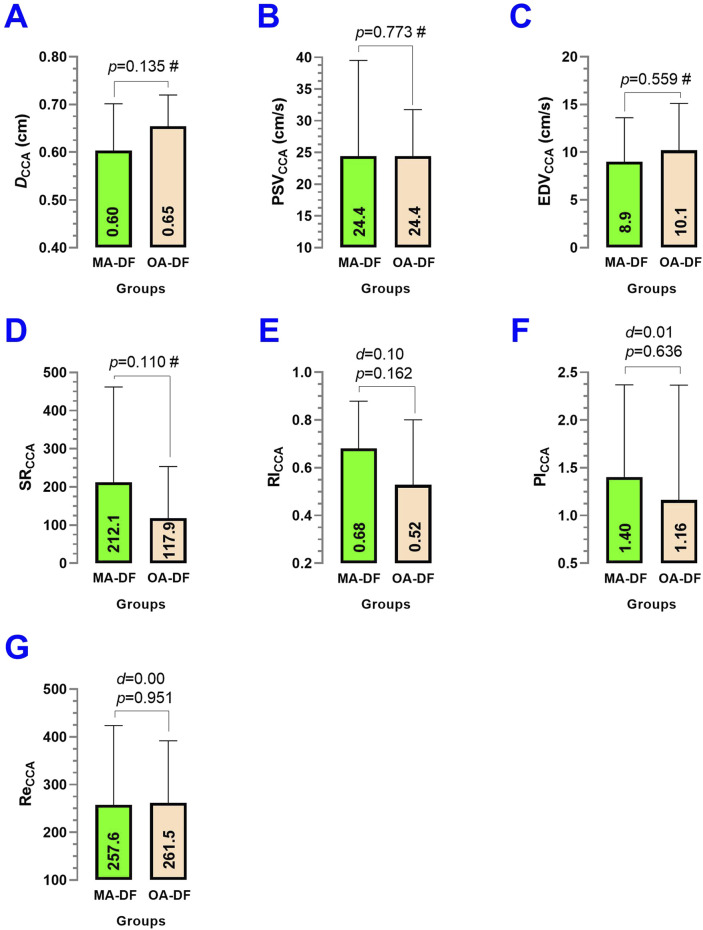
Common carotid artery diameter (*D*_CCA_) **(A)**, peak systolic velocity (PSV_CCA_) **(B)**, end-diastolic velocity (EDV_CCA_) **(C)**, shear rate (SR_CCA_) **(D)**, resistance index (RI_CCA_) **(E)**, pulsatility index (PI_CCA_) **(F)**, and Reynolds number (Re_CCA_) **(G)** in diver fishermen of the southern Chilean Coast dedicated to mollusk harvesting. Groups are described as middle-aged diver fishermen (MA-DF) and older adult diver fishermen (OA-DF). (#) Analyzed by an unpaired *t*-test at *P* < 0.05. (*d*) Cohen’s *d* effect size at *p* < 0.05.

In the CCA, comparing MA-DF vs. OA-DF, the *D*_CCA_ (*p* = 0.135) (Figure 4A), PSV_CCA_ (*p* = 0.773) (Figure 4B), EDV_CCA_ (*p* = 0.559) (Figure 4C), SR_CAA_ (*p* = 0.110) (Figure 4D), RI_BA_ (*p* = 0.162) (Figure 4E), PI_CCA_ (*p* = 0.636) (Figure 4F), and Re_CCA_ (*p* = 0.951) (Figure 4G) showed no significant differences.

### 3.3 Arterial stiffness, intima–media thickness, and covariates characteristics

**FIGURE 5:**
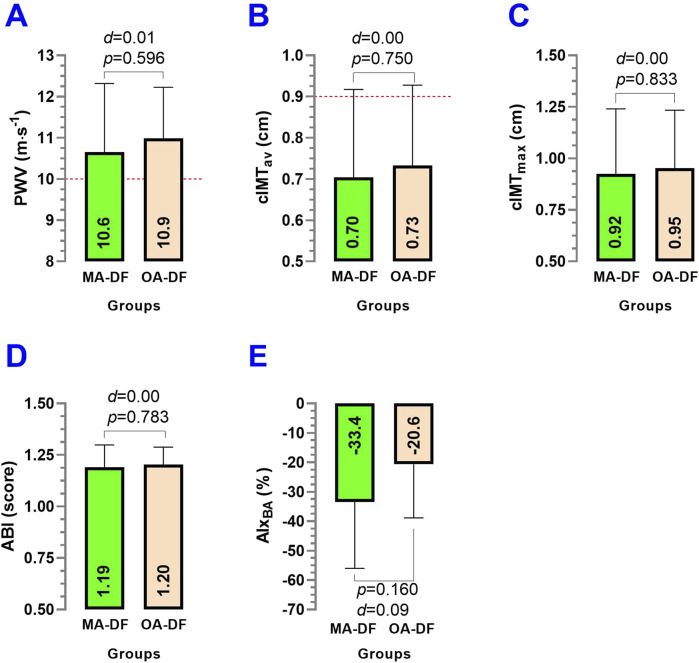
Pulse wave velocity (PWV) **(A)**, carotid intima–media thickness average (cIMT_av_) **(B)**, carotid intima–media thickness maximum (cIMT_max_) **(C)**, ankle-brachial index (ABI) **(D)**, and augmentation index of the brachial artery (AIx_BA_) **(E)** of the common carotid artery in diver fishermen of the southern Chilean Coast dedicated to mollusk harvesting. Groups are described as middle-aged diver fishermen (MA-DF) and older adult diver fishermen (OA-DF). (#) Analyzed by the unpaired *t*-test at *p* < 0.05. (*d*) Cohen’s *d* effect size at *p* < 0.05.

Comparing MA-DF vs. OA-DF, the PWV (*p* = 0.596) (Figure 5A), cIMT_av_ (*p* = 0.750) (Figure 5B), cIMT_max_ (*p* = 0.833) (Figure 5C), ABI (*p* = 0.783) (Figure 5D), and AIx_BA_ (*p* = 0.160) (Figure 5E) showed no significant differences between groups.

### 3.4 Physical fitness characteristics

**FIGURE 6:**
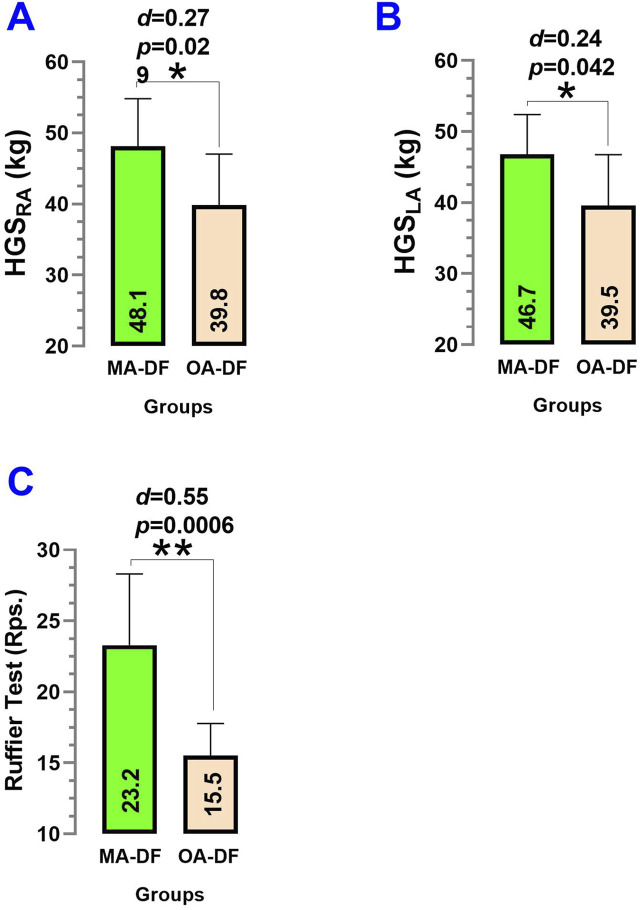
Handgrip strength right arm **(A)**, handgrip strength left arm **(B)**, and Ruffier test of 45 s “squat” exercise **(C)** in diver fishermen of the southern Chilean Coast dedicated to mollusk harvesting. Groups are described as middle-aged diver fishermen (MA-DF) and older adult diver fishermen (OA-DF). (*) denotes significant differences by unpaired *t*-test at *p* < 0.05. (**) denotes significant differences by unpaired *t*-test at *p* < 0.001. (*d*) Cohen’s *d* effect size at *p* < 0.05.

Comparing MA-DF vs. OA-DF, there were significant differences in outcomes HGS_RA_ (48.1 ± 6.2 kg vs. 39.8 ± 6.4 kg, *p* = 0.029, *d* = 0.27) (Figure 6A), HGS_LA_ (46.7 ± 5.9 kg vs. 39.5 ± 6.3 kg, *p* = 0.042, *d* = 0.24) (Figure 6B), and in the Ruffier test repetitions performed in the 45 s squat exercise Ruffier test (23.2 ± 5.3 repetitions vs. 15.5 ± 2.4 repetitions, *p* = 0.0006) (Figure 6C).

### 3.5 Lifestyle characteristics

**FIGURE 7:**
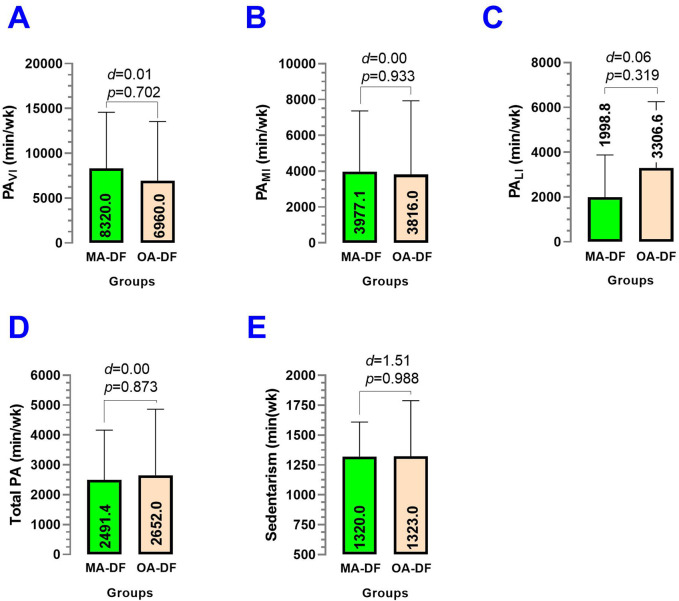
Physical activity of vigorous **(A)**, moderate **(B)**, and light intensity **(C)**, total physical activity **(D)**, and sedentary behavior **(E)** in diver fishermen of the southern Chilean Coast dedicated to mollusk harvesting. Groups are described as middle-aged diver fishermen (MA-DF) and older adult diver fishermen (OA-DF). (*d*) Cohen’s d effect size at *p* < 0.05.

Comparing MA-DF vs. OA-DF, the vigorous PA_VI_ (*p* = 0.702) (Figure 7A), moderate PA_MI_ (*p* = 0.933) (Figure 7B), light physical activity PA_LI_ (*p* = 0.319) (Figure 7C), total physical activity (*p* = 0.873) (Figure 7D), and sedentary behavior per week (*p* = 0.988) (Figure 7E) showed no significant differences between groups.

### 3.6 Body fat characteristics

**FIGURE 8:**
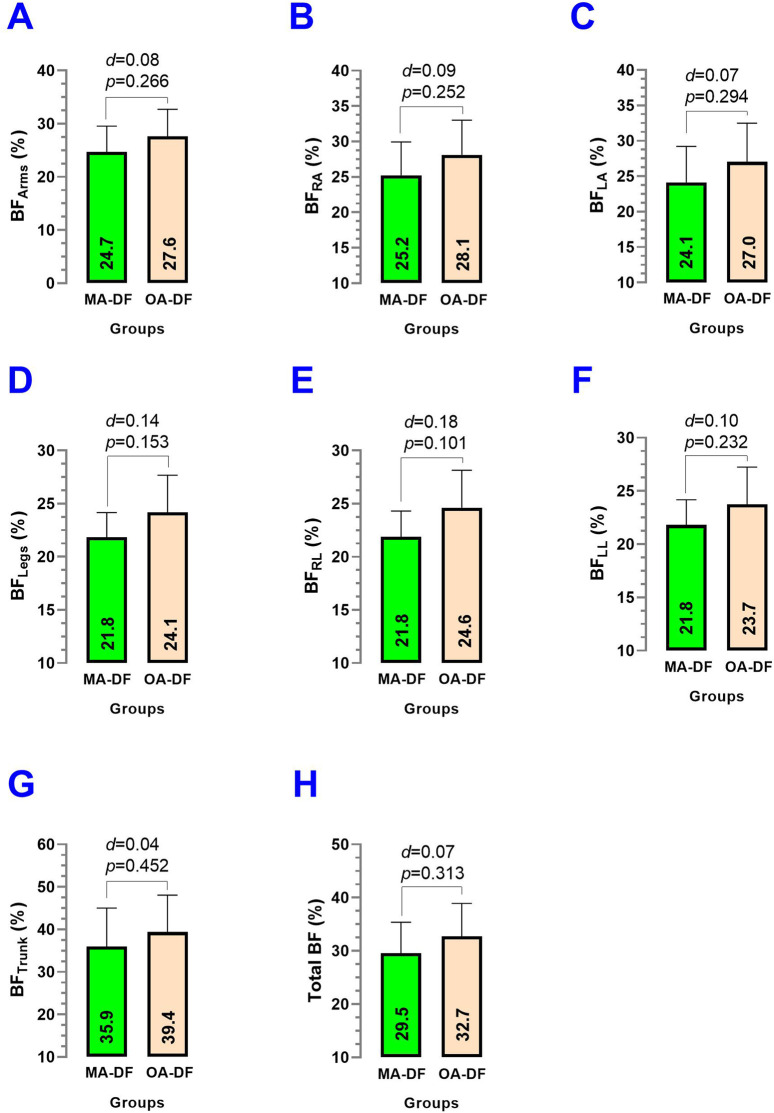
Body fat of the arms **(A)**, body fat of the right arm **(B)**, body fat of the left arm **(C)**, body fat of the legs **(D)**, body fat of the right leg **(E)**, body fat of the left leg **(F)**, body fat of the trunk **(G)**, and total body fat % **(H)** in diver fishermen of the southern Chilean Coast dedicated to mollusk harvesting. Groups are described as middle-aged diver fishermen (MA-DF) and older adult diver fishermen (OA-DF).

Comparing MA-DF vs. OA-DF, the % body fat in arms BF_Arms_ (*p* = 0.266) (Figure 8A), % body fat of the left arm BF_LA_ (*p* = 0.294) (Figure 8B), body fat of the right arm BF_RA_ (*p* = 0.252) (Figure 8C), % body fat legs BF_Legs_ (*p* = 0.153) (Figure 8D), % body fat right leg BF_RL_ (*p* = 0.101) (Figure 8E), % body fat left leg BF_LL_ (*p* = 0.232) (Figure 8F), % body fat trunk BF_T_ (*p* = 0.452) (Figure 8G), and total body fat % Total BF (*p* = 0.313) (Figure 8H) showed numerically higher values in the MA-DF group but were non-significant differences (*p* > 0.05).

### 3.7 Fat-free mass characteristics

**FIGURE 9:**
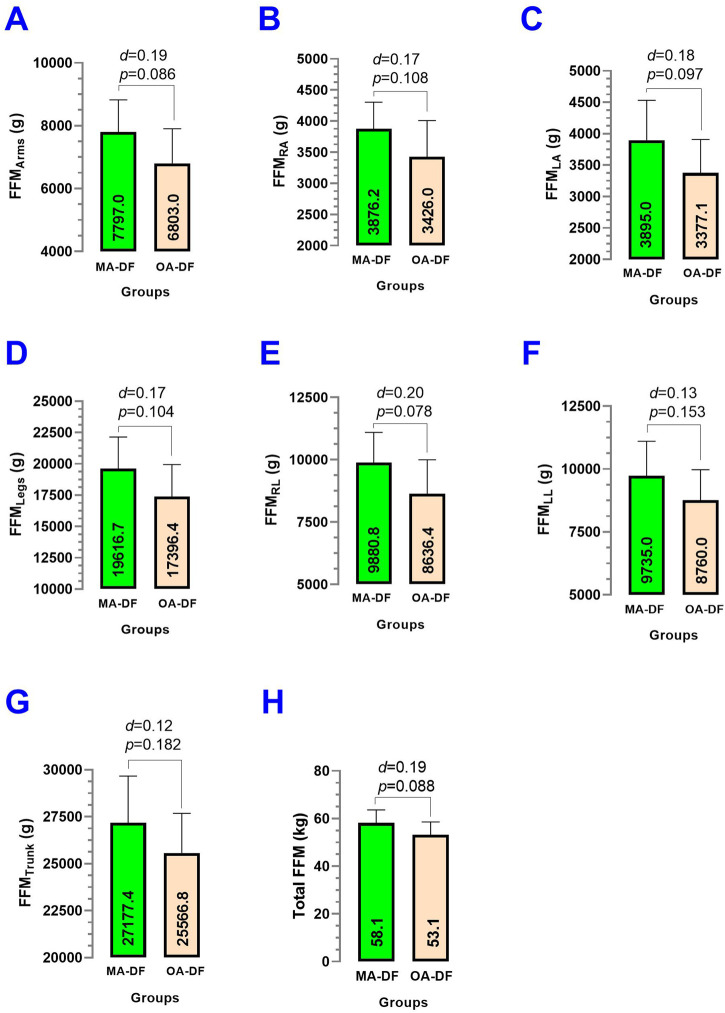
Fat-free mass of the arms **(A)**, fat-free mass of the right arm **(B)**, fat-free mass of the left arm **(C)**, fat-free mass of the legs **(D)**, fat-free mass of the right leg **(E)**, fat-free mass of the left leg **(F)**, fat-free mass of the trunk **(G)**, and total fat-free mass **(H)** in diver fishermen of the southern Chilean Coast dedicated to mollusk harvesting. Groups are described as middle-aged diver fishermen (MA-DF) and older adult diver fishermen (OA-DF). (*d*) Cohen’s *d* effect size at *p* < 0.05.

There were no significantly different (*p* > 0.05) when comparing MA-DF vs. OA-DF groups in terms of fat-free mass in arms FFM_A_ (*p* = 0.086) (Figure 9A), fat-free mass in right arm FFM_RA_ (*p* = 0.108) (Figure 9B), fat-free mass in left arm FFM_LA_ (*p* = 0.097) (Figure 9C), fat-free mass legs FFM_L_ (*p* = 0.104) (Figure 9D), fat-free mass right leg FFM_RL_ (*p* = 0.078) (Figure 9E), fat-free mass left leg FFM_LL_ (*p* = 0.153) (Figure 9F), fat-free mass trunk FFM_T_ (*p* = 0.182) (Figure 9G), and total fat-free mass Total FFM (*p* = 0.088) (Figure 9H).

### 3.8 Bone mineral content characteristics

**FIGURE 10:**
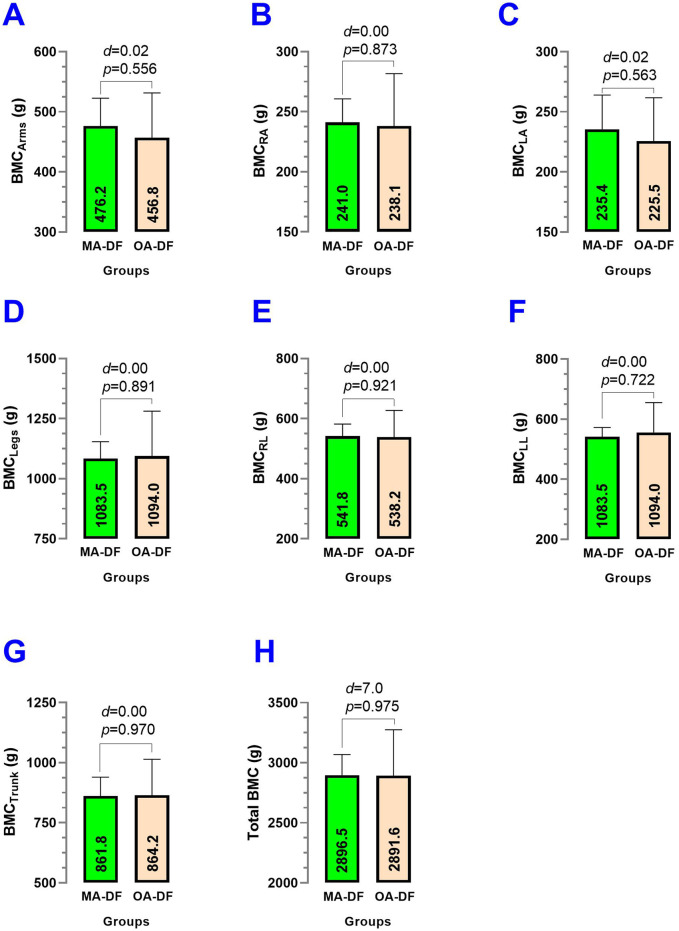
Bone mineral content (BMC) of the arms **(A)**, BMC of the right arm **(B)**, BMC of the left arm **(C)**, BMC of the legs **(D)**, BMC of the right leg **(E)**, BMC of the left leg **(F)**, BMC of the trunk **(G)**, and total BMC **(H)** in diver fishermen of the southern Chilean Coast dedicated to mollusk harvesting. Groups are described as middle-aged diver fishermen (MA-DF) and older adult diver fishermen (OA-DF).

Comparing MA-DF vs. OA-DF, the bone mineral content in arms BMC_Arms_ (*p* = 0.556) (Figure 10A), bone mineral content in right arm BMC_RA_ (*p* = 0.873) (Figure 10B), bone mineral content in left arm BMC_LA_ (*p* = 0.563) (Figure 10C), bone mineral content legs BMC_Legs_ (*p* = 0.891) (Figure 10D), bone mineral content right leg BMC_RL_ (*p* = 0.921) (Figure 10E), bone mineral content left leg BMC_LL_ (*p* = 0.722) (Figure 10F), bone mineral content trunk BMC_T_ (*p* = 0.970) (Figure 10G), and total bone mineral content Total BMC (*P* = 0.975) (Figure 10H) showed no significant differences between groups (*p* > 0.05).

### 3.9 Segmental body composition differences between groups

**FIGURE 11:**
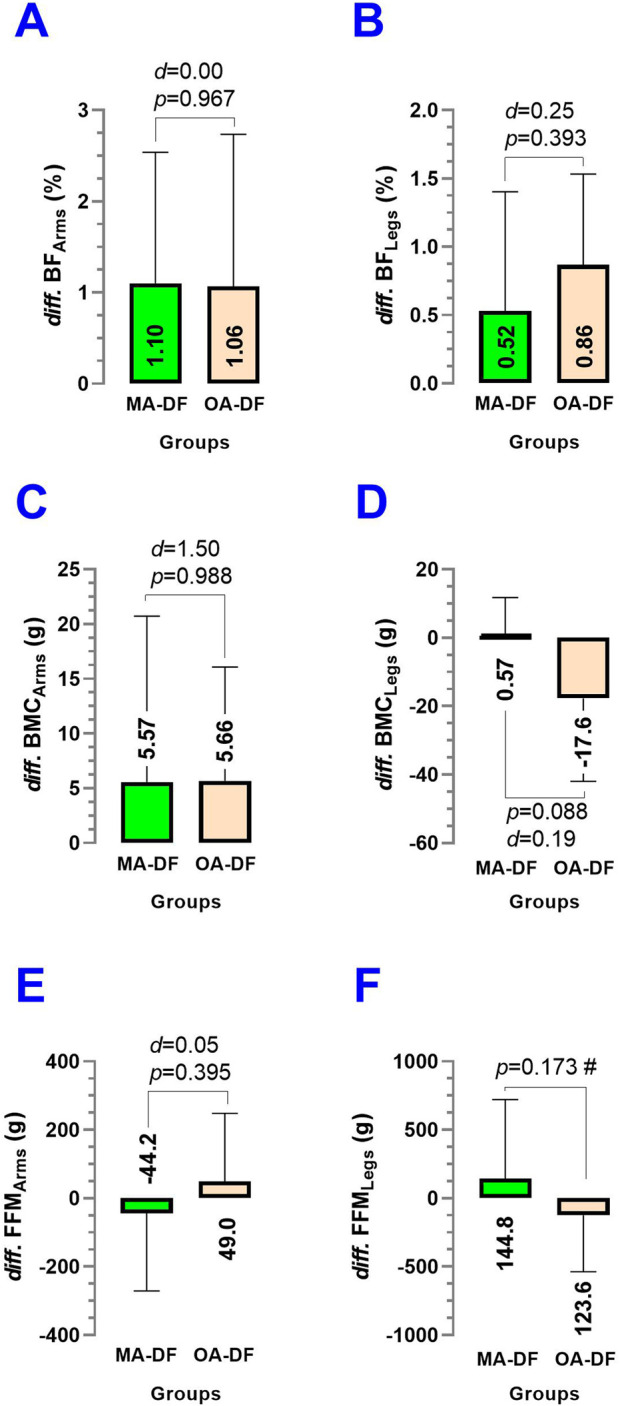
Differences (*diff*) between the body fat % of the arms **(A)**, *diff*. of the legs **(B)**, *diff*. of the bone mineral content (BMC) of the arms **(C)**, *diff*. of the BMC of the legs **(D)**, *diff*. of the fat-free mass [FFM] of the arms **(E)**, and diff. of the FFM of the legs **(F)** in diver fishermen of the southern Chilean Coast dedicated to mollusk harvesting. Groups are described as middle-aged diver fishermen (MA-DF) and older adult diver fishermen (OA-DF).

The segmental differences in body composition comparing MA-DF vs. OA-DF in outcomes *diff.* BF_Arms_ (*p* = 0.967) (Figure 11A), *diff.* BF_Legs_ (*p* = 0.393) (Figure 11B), *diff.* BMC_Arms_ (*p* = 0.988) (Figure 11C), *diff.* BMC_Legs_ (*p* = 0.088) (Figure 11D), FFM_Arms_ (*p* = 0.395), and FFM_Legs_ (*p* = 0.173) showed no significant differences between groups (*p* > 0.05).

### 3.10 Association between physical fitness and blood flow characteristics

For the BA structure, no significant associations were observed between *D*_BA_ and age, MAP, HGS_AV_, or Ruffier test performance (all p > 0.05). Regarding BA function, Ruffier test performance showed a strong and significant positive association with SR_BA_ (B = 88.7 s^−1^ per repetition, *p* = 0.003, 95%CI: 35.8; 141.9), whereas age, MAP, and HGS_AV_ were not significantly associated with SR_BA_ (all *p* > 0.05). For the CCA structure, *D*_CCA_ was not significantly associated with age, MAP, HGS_AV_, or Ruffier test performance (all *p* > 0.05) (Table 2). In terms of CCA function, Ruffier test performance was significantly and positively associated with SR_CCA_ (B = 38.7 s^−1^ per repetition, *p* = 0.042, 95%CI: 1.5; 75.8). No significant associations were found between SR_CCA_ and age, MAP, or HGS_AV_ (Table 2).

**TABLE 2:** Association of vascular outcomes related to the brachial and common carotid artery structure and function and upper- and lower-limb physical fitness in Latin American diver fishermen of the Chilean coast.

Dependent outcome—predictor	B (estimate)	SE	*t-*test	*p-*value	95%CI
*Brachial artery structure*					
Intercept	**10.4**	**4.2**	**2.4**	***p* = 0.028**	**1.2; 19.6**
*D*_BA_ (mm) – ^Adj.^Age (y)	−0.03	0.03	1.2	*p* = 0.230	−0.1; 0.02
*D*_BA_ (mm) – ^Adj.^MAP (mmHg)	−0.01	0.02	0.6	*p* = 0.542	−0.06; 0.03
*D*_BA_ (mm) – HGS_AV_ (kg)	−0.01	0.02	0.2	*p* = 0.777	−0.09; 0.06
*D*_BA_ (mm) – Ruffier test (repetitions)	−0.08	0.06	1.3	*p* = 0.211	−0.2; 0.05
*Brachial artery function*					
Intercept	−2,342.0	1,655.0	1.4	*p* = 0.182	−5,948.0; 1,264.0
SR_BA_^(s-1)^ – ^Adj.^Age (y)	13.9	11.8	1.1	*p* = 0.258	−11.7; 39.7
SR_BA_^(s-1)^ – ^Adj.^MAP (mmHg)	6.4	8.6	0.7	*p* = 0.468	−12.3; 25.3
SR_BA_^(s-1)^ – HGS_AV_ (kg)	−14.9	14.4	1.0	*p* = 0.320	−46.3; 16.4
SR_BA_^(s-1)^ – Ruffier test (repetitions)	**88.7**	**24.3**	**3.6**	***p* = 0.003**	**35.8; 141.9**
*Common carotid artery structure*					
Intercept	0.9	0.4	2.0	*p* = 0.067	−0.07; 1.8
*D*_CCA_ (mm) – ^Adj.^Age (y)	−0.0001	0.003	0.04	*p* = 0.963	−0.007; 0.006
*D*_CCA_ (mm) – ^Adj.^MAP (mmHg)	−0.0008	0.002	0.3	*p* = 0.730	−0.005; 0.004
*D*_CCA_ (mm) – HGS_AV_ (kg)	−0.003	0.003	1.0	*p* = 0.329	−0.01; 0.004
*D*_CCA_ (mm) – Ruffier test (repetitions)	0.0007	0.006	0.1	*p* = 0.913	−0.01; 0.01
*Common carotid artery function*					
Intercept	−658.3	1,217.0	0.5	*p* = 0.599	−3,338.0; 2021.0
SR_CCA_^(s-1)^ – ^Adj.^Age (y)	3.3	8.4	0.3	*p* = 0.699	−15.3; 22.0
SR_CCA_^(s-1)^ – ^Adj.^MAP (mmHg)	3.0	6.0	0.4	*p* = 0.627	−10.3; 16.3
SR_CCA_^(s-1)^ – HGS_AV_ (kg)	−10.3	10.0	1.0	*p* = 0.326	−32.5; 11.8
SR_CCA_^(s-1)^ – Ruffier test (repetitions)	**38.7**	**16.8**	**2.2**	***p* = 0.042**	**1.5; 75.8**

Outcomes are described as brachial artery diameter (*D*_BA_), mean arterial pressure (MAP), handgrip strength average of right and left arms (HGS_AV_), common carotid artery diameter (*D*_CCA_), shear rate brachial artery (SR_BA_), shear rate common carotid artery (SR_CCA_), 95% confidence interval (95%CI), and adjusted variables to the regression model (Adj.). Bold values denote significant associations in multivariable linear regression analyses at *p* < 0.05.

## 4 Discussion

The primary aim of the present study was to describe the vascular, physical fitness, lifestyle, and body composition characteristics of middle-aged and older adult diver fishermen. The second aim was to associate vascular outcomes with the physical fitness of the upper and lower limbs. Thus, the present study has three main results: *i*) middle-aged and older adult DF show similar vascular (functional and structural), lifestyle, and body composition characteristics; *ii*) significant differences in upper- and lower-limb physical fitness favored the middle-aged DF; and *iii*) a significant association was found between the vascular parameters of function SR_BA_, and SR_CCA_ with lower-limb physical fitness (i.e., by the Ruffier test repetitions) (Table 2). Overall, the present results suggest that lower-limb physical fitness evaluated using the Ruffier test is independently associated with peripheral (SR_BA_) and central vascular outcomes of function (SR_CCA_), whereas no association was observed with arterial structural variables of diameters, after controlling for relevant confounders. These findings suggest that enhanced lower-limb functional capacity is positively associated with more favorable flow dynamics and vascular health in peripheral (i.e., BA) and central (i.e., CCA) arteries in DF (Table 2), which may help inform and support the development of specific lifestyle strategies to promote adequate physical fitness in active divers.

About our first result, the observation that the BA (*D*_BA_, PSV_BA_, EDV_BA_, SR_BA_, RI_BA_, PI_BA_, and Re_BA_) (Figure 3), carotid (*D*_CCA_, PSV_CCA_, EDV_CCA_, SR_CCA_, RI_CCA_, PI_CCA_, and Re_CCA_) (Figure 4), and PWV, cIMT_av_, cIMT_max_, ABI, AIx_BA_ vascular parameters did not differ significantly between middle-aged and older adult DF could suggest a preserved vascular health in this occupational group, despite almost ∼20 years of chronological age difference. Worryingly, the similarity in PWV in both MA-DF and OA-DF was higher than 10 m·s^−1^, which denotes a high CVD risk in both MA-DF and OA-DF groups (Figure 5A). At the same time, cIMT_av_ was apparently exceeded in SD in some participants, as shown in Figure 5B. Thus, independent of biological aging, both MA-DF and OA-DF may need additional PA/exercise practice and to improve their lifestyle conditions (*i.e.,* PA patterns) to decrease arterial stiffness and CVD risk, as has been shown in previous studies (Vogel et al., 2013; Alvarez et al., 2024). Vogel et al. reported that 9 weeks of intermittent endurance exercise training in healthy adults decreased PWV by −0.6 m·s^−1^. We previously reported that 6 weeks of exercise training decreased PWV by −1.2 m·s^−1^ in hypertensive subjects under additional obesity conditions (Alvarez et al., 2024). Thus, these early vascular risks in MA-DF and OA-DF could be potentially treated with lifestyle interventions.

About our second result, the analysis of physical fitness outcomes revealed significantly greater HGS_RA_ and HGS_LA_ and a higher number of repetitions during the Ruffier squat test in the middle-aged group (Figure 6). These differences likely reflect age-related declines in muscular strength (i.e., upper limb) and functional capacity (i.e., lower limb), consistent with prior findings in aging populations. However, it is notable that these older adult divers stay physically active and keep moderate to high physical activity levels, which may mitigate some functional losses. Thus, we presume that despite no lifestyle differences between MA-DF vs. OA-DF in lifestyle PA_VI_, PA_MI_, PA_LI_, it is important to promote more structured and specific PA and exercise regimes, such as resistance training including external weights, in these populations of active occupational activity during older adulthood to promote an increase in skeletal muscle mass and thus avoid some early symptoms of chronological aging. For example, Cavani et al. (2002) reported that after 6 weeks of resistance training plus stretching exercise (3 sessions/week, older adult participants (∼70 years) improved their performance during the Ruffier test. A systematic review and meta-analysis of Radaelli et al. (2025) with 151 randomized trials summarized that short (i.e., ≤20 weeks) and middle-term resistance training programs (i.e., ≥20 weeks) can significantly increase muscle size in older adults. Another recent systematic review from Khaleghi et al. (2025) summarizing different water sports reported that short interventions (i.e., including swimming and diving) of ∼14 days show potential for cardiovascular health by improving body composition and physical fitness.

Our third key finding of this study was the significant association between flow parameters of vascular function (SR_BA_ and SR_CCA_) with the Ruffier test repetitions (Table 2). These associations suggest a strong link between dynamic muscular activity of the lower limbs with peripheral and central arterial hemodynamics, reinforcing the concept that physical fitness, evaluated from a large muscle mass in the form of a lower-limb squat exercise, can modulate vascular function even in older adult individuals. Jun et al. (2021) reported recently in a sample of *n* = 5,401 followed for 2 years that the lowest skeletal muscle mass index quartile [calculated by: total appendicular muscle mass (kg)/body weight (kg) × 100] was significantly associated with the presence of increased risk of coronary artery calcification in adults ∼50 years old. In contrast, upper limb strength, assessed by HGS_AV_, was not associated with vascular parameters in either BA or CCA (Table 2). This may indicate that the type of muscular activity represented by handgrip strength could not sufficiently stimulate vascular adaptations, or that its effects on vascular blood fluxes and hemodynamics are less systemic than those induced by lower-limb endurance activities that involve greater skeletal muscle mass.

Secondary outcomes, such as lifestyle (PA_VI_, PA_MI_, PA_LI_, sedentary time/wk), body composition (BF_Arms_, BF_RA_, BF_LA_, BF_Legs_, BF_RL_, BF_LL_, BF_Trunk_, Total BF; FFM_Arms_, FFM_RA_, FFM_LA_, FFM_Legs_, FFM_RL_, FFM_LL_, FFM_Trunk_, Total FFM; and BMC_Arms_, BMC_RA_, BMC_LA_, BMC_Legs_, BMC_RL_, BMC_LL_, BMC_Trunk_, and Total BMC), were similar between groups. This suggests that observed differences in vascular health are unlikely to be driven by these variables and further emphasizes the role of physical fitness in influencing vascular dynamics. On the other hand, despite our precision iDXA body composition measurements and no significant statistical differences between groups, there was a trend that BF% was higher (Figure 8), but FFM and BMC outcomes were slightly lower in OA-DF than MA-DF (Figures 9, 10). However, considering the active diving condition of the OA-DF group, it is also relevant to promote more specific lifestyle strategies in this group. Examples include reinforcing adherence to the international physical activity guidelines or specific strength training for maintenance of the FFM, muscle mass, and BMC to avoid potential frailty conditions and also to decrease body fat content to reduce the risk for CVD. Interestingly, when we compared the MA-DF and OA-DF groups in terms of the segmental arms or legs analysis, the BMC_Legs_ outcome was almost a significant difference (*diff*.) between the groups (*p* = 0.088, Figure 11), meaning that older adult DF are at more risk for experiencing balanced bone demineralization between legs with potential consequences to functional capacity and locomotion. From here, we presume that the nature of our pilot study in terms of low sample size can be further clarified in future descriptive or cross-sectional studies with more robust samples.

Overall, these results highlight the importance of maintaining lower-limb physical fitness (i.e., particularly functional capacity) in older-age occupational groups of major longevity like DF, who need more lifestyle interventions to support both vascular and musculoskeletal health. Recently, the Chilean Superintendence of Social Security reported that 36% of DF report a smoking habit, ∼4–5% report bone fractures as the most common type of accident, and 86.7% show overweight/obesity. These findings increase the need for more robust studies (SUSESO, 2020). The predictive associations between Ruffier test “squat” performance and the vascular outcome SR underscore its potential utility as a practical screening tool in aging DF workers exposed to unique physical environments in the waters of the Chilean coast.

### 4.1 Limitations and strengths

This study has several limitations. First, the sample size was small, and participants were recruited voluntarily, which may limit generalizability. Second, heart rate during the Ruffier test was measured manually, which could introduce variability. Third, potential environmental factors such as seasonal diving patterns and water temperature were not considered. Fourth, the seasons with longer or shorter frequency of diving periods were not quantified. Fifth, as with any study of an associative nature, these findings do not imply a cause–effect relationship; therefore, future studies with greater methodological complexity are needed to corroborate these preliminary results. Sixth, future applications of the Ruffier test should consider using a heart rate monitor to record this parameter more objectively. Despite these limitations, the study also has notable strengths, including *i*) it focused on a difficult-to-reach occupational population; *ii*) it employed gold-standard iDXA for body composition analysis; *iii*) it includes individuals across a wide range of chronological ages, providing a more comprehensive characterization of the physiological traits of this population, which typically remains active in this occupational context well into advanced age; *iv*) it provided novel insights into the relationship between physical fitness and vascular parameters in aging divers; *v*) all the measurements taken are part of a preventive plan with social authorities, which will allow future health promotion coordination to be proposed from the research team.

## 5 Conclusion

Despite similar vascular, lifestyle, and body composition profiles, middle-aged and older diver fishermen displayed marked differences in upper- and lower-limb physical fitness. Importantly, lower-limb physical fitness, as assessed by the Ruffier test, emerged as a robust correlate of vascular SR in both the BA and CCA, highlighting its potential relevance to peripheral and central vascular function.

## Data Availability

The raw data supporting the conclusions of this article will be made available by the authors, without undue reservation.

## References

[B1] Alahmari K. A. Rengaramanujam K. Reddy R. S. Samuel P. S. Kakaraparthi V. N. Ahmad I. (2020). Cardiorespiratory fitness as a correlate of cardiovascular, anthropometric, and physical risk factors: using the ruffier test as a template. Can. Respir. J. 2020 (1), 3407345. 10.1155/2020/3407345 32963643 PMC7495241

[B26] Armada de Chile (2014). Reglamento de Buceo para Buzos Profesionales (TM-035). Valparaíso: Dirección General del Territorio Marítimo y de Marina Mercante. Available online at: https://www.directemar.cl/directemar/site/docs/20170308/20170308093133/tm_035.pdf?utm_source=chatgpt.com.

[B2] Álvarez C. Peñailillo L. Saavedra P. I. Roa M. T. Mayorga D. A. J. Domaradski J. (2024). Exercise training is effective for arterial stiffness and blood pressure rehabilitation in hypertensive adults. Retos nuevas tendencias Educ. física, deporte recreación (56), 301–311. 10.47197/retos.v56.104740

[B3] Alvarez C. Peñailillo L. Ibacache-Saavedra P. Jerez-Mayorga D. Campos-Jara C. Andrade D. C. (2024). Six weeks of a concurrent training therapy improves endothelial function and arterial stiffness in hypertensive adults with minimum non-responders. Hipertens. Y Riesgo Vasc. 41 (4), 240–250. 10.1016/j.hipert.2024.07.001 39079872

[B4] Bevier W. C. Wiswell R. A. Pyka G. Kozak K. C. Newhall K. M. Marcus R. (1989). Relationship of body composition, muscle strength, and aerobic capacity to bone mineral density in older men and women. J. Bone Mineral Res. 4 (3), 421–432. 10.1002/jbmr.5650040318 2763878

[B5] Bove A. A. (2014). Diving medicine. Am. Journal Respiratory Critical Care Medicine 189 (12), 1479–1486. 10.1164/rccm.201309-1662CI 24869752

[B6] Cavani V. Mier C. M. Musto A. A. Tummers N. (2002). Effects of a 6-Week resistance-training program on functional fitness of older adults. J. Aging Phys. Activity 10 (4), 443–452. 10.1123/japa.10.4.443

[B7] Coll B. Feinstein S. B. (2008). Carotid intima-media thickness measurements: techniques and clinical relevance. Curr. Atherosclerosis Reports 10 (5), 444–450. 10.1007/s11883-008-0068-1 18706287

[B8] Dormanesh B. Vosoughi K. Akhoundi F. H. Mehrpour M. Fereshtehnejad S.-M. Esmaeili S. (2016). Carotid duplex ultrasound and transcranial doppler findings in commercial divers and pilots. Neurol. Sci. 37 (12), 1911–1916. 10.1007/s10072-016-2674-y 27461112

[B9] Gomez M. Montalvo S. Lozano A. Arias S. Gurovich A. N. (2022). Brachial artery blood flow patterns during eccentric cycling exercise: 164. Med. and Sci. Sports and Exerc. 54 (9S), 32–33. 10.1249/01.mss.0000875460.77098.0c

[B10] González-García I. McCarthy H. D. (2022). An evaluation of the association between anthropometric measurements and cardiorespiratory fitness using the forest service step and the ruffier-dickson test. Med. Dello Sport 75 (3), 391–403. 10.23736/S0025-7826.22.04044-3

[B11] Guatibonza-García V. Gnecco-González S. Pérez-Londoño A. Betancourt-Villamizar C. Mendivil C. O. (2025). A descriptive study of smoking, socioeconomic position, and health-related behaviors in urban Colombia. Discov. Public Health 22 (1), 22. 10.1186/s12982-025-00405-z

[B12] Hwang H. Bae J. Hwang S. Park H. Kim I. (2006). Effects of breath-hold diving on bone mineral density of women divers. Jt. bone, spine Rev. Du. Rhum. 73 (4), 419–423. 10.1016/j.jbspin.2005.07.005 16626996

[B13] Joussellin E. (2007). Filetest de Ruffier, improprement appelé test de Ruffier-Dickson. Med. Du. Sport 83 (4), 33–34.

[B14] Jun J. E. Choi M. S. Park S. W. Kim G. Jin S.-M. Kim K. (2021). Low skeletal muscle mass is associated with the presence, incidence, and progression of coronary artery calcification. Can. J. Cardiol. 37 (9), 1480–1488. 10.1016/j.cjca.2021.04.002 33845138

[B15] Khaleghi M. M. Zar A. Krustrup P. Al Kitani M. (2025). Effects of water sports on heart disease risk factors: a systematic review. Sport Sci. Health 21 (3), 1337–1348. 10.1007/s11332-025-01379-w

[B16] Lazić A. Danković G. Korobeinikov G. Cadenas-Sanchez C. Trajković N. (2025). Acute effects of different “exercise snacking”modalities on glycemic control in patients with type 2 diabetes mellitus (T2DM): study protocol for a randomized controlled trial. BMC Public Health 25 (1), 566. 10.1186/s12889-025-21669-9 39934727 PMC11817963

[B17] Li H. Wang H. (2025). Association between weekend warrior physical activity pattern and bone mineral density among adults: national health and nutrition examination survey. Osteoporos. Int. 36 (7), 1221–1229. 10.1007/s00198-025-07535-9 40418339

[B18] Mancia G. Fagard R. Narkiewicz K. Redón J. Zanchetti A. Böhm M. (2013). 2013 ESH/ESC guidelines for the management of arterial hypertension: the task force for the management of arterial hypertension of the european society of hypertension (ESH) and of the european society of cardiology (ESC). J. Hypertens. 31 (7), 1281–1357. 10.1097/01.hjh.0000431740.32696.cc 23817082

[B19] Marfell-Jones M. Olds T. Stewart A. Carter L. (2006). International standards for anthropometric assessment.

[B20] Marx N. Federici M. Schütt K. Müller-Wieland D. Ajjan R. A. Antunes M. J. (2023). 2023 ESC guidelines for the management of cardiovascular disease in patients with diabetes: developed by the task force on the management of cardiovascular disease in patients with diabetes of the european society of cardiology (ESC). Eur. Heart J. 44 (39), 4043–4140. 10.1093/eurheartj/ehad192 37622663

[B21] Natale F. Ranieri A. Siciliano A. Casillo B. Di Lorenzo C. Granato C. (2014). Rapid ultrasound score as an indicator of atherosclerosis' clinical manifestations in a population of hypertensives: the interrelationship between flow-mediated dilatation of brachial artery, carotid intima thickness, renal resistive index and retina resistive index of central artery. Anatol. J. Cardiology/Anadolu Kardiyoloji Dergisi. 14 (1), 9–15. 10.5152/akd.2013.4823 24342928

[B22] Obad A. Marinovic J. Ljubkovic M. Breskovic T. Modun D. Boban M. (2010). Successive deep dives impair endothelial function and enhance oxidative stress in man. Clin. Physiology Functional Imaging 30 (6), 432–438. 10.1111/j.1475-097X.2010.00962.x 20718805

[B23] Perovic A. Nikolac N. Braticevic M. N. Milcic A. Sobocanec S. Balog T. (2017). Does recreational scuba diving have clinically significant effect on routine haematological parameters? Biochem. Med. Zagreb. 27 (2), 325–331. 10.11613/BM.2017.035 28694723 PMC5493166

[B24] Piquet L. Dalmay F. Ayoub J. Vandroux J. C. Menier R. Antonini M. T. (2000). Study of blood flow parameters measured in femoral artery after exercise: correlation with maximum oxygen uptake. Ultrasound Med. Biol. 26 (6), 1001–1007. 10.1016/s0301-5629(00)00222-2 10996700

[B25] Radaelli R. Rech A. Molinari T. Markarian A. M. Petropoulou M. Granacher U. (2025). Effects of resistance training volume on physical function, lean body mass and lower-body muscle hypertrophy and strength in older adults: a systematic review and network meta-analysis of 151 randomised trials. Sports Med. 55 (1), 167–192. 10.1007/s40279-024-02123-z 39405023

[B27] Ring M. Eriksson M. J. Zierath J. R. Caidahl K. (2014). Arterial stiffness estimation in healthy subjects: a validation of oscillometric (arteriograph) and tonometric (SphygmoCor) techniques. Hypertens. Res. 37 (11), 999–1007. 10.1038/hr.2014.115 25056681

[B28] Santos C. A. Maia H. F. Pitanga F. J. G. de Almeida M. C. C. da Fonseca M. J. M. de Aquino E. M. L. (2025). Hand grip strength cut‐off points as a discriminator of sarcopenia and sarcopenic obesity: results from the ELSA‐Brasil cohort. J. Cachexia, Sarcopenia Muscle 16 (1), e13723. 10.1002/jcsm.13723 39966694 PMC11835543

[B29] Sartor F. Bonato M. Papini G. Bosio A. Mohammed R. A. Bonomi A. G. (2016). A 45-second self-test for cardiorespiratory fitness: heart rate-based estimation in healthy individuals. PloS One 11 (12), e0168154. 10.1371/journal.pone.0168154 27959935 PMC5154562

[B30] Seth C. Schmid V. Mueller S. Haykowsky M. Foulkes S. J. Halle M. (2025). Diabetes, obesity, and cardiovascular disease—what is the impact of lifestyle modification? Herz 50, 240–245. 10.1007/s00059-025-05309-x 40085207

[B31] Souto Cavalli L. Tapia-Jopia C. Ochs C. López Gómez M. A. Neis B. (2023). Salmon mass mortality events and occupational health and safety in Chilean aquaculture. All Life. 16 (1), 2207772. 10.1080/26895293.2023.2207772

[B32] Subramanian V. Tucker W. J. Peters A. E. Upadhya B. Kitzman D. W. Pandey A. (2025). Cardiovascular aging and exercise: implications for heart failure prevention and management. Circulation Res. 137 (2), 205–230. 10.1161/CIRCRESAHA.125.325531 40608862

[B33] SUSESO (Superintendencia de Seguridad Social) (2020). Estudio observacional de buzos dedicados a la acuicultura, año 2017. Available online at: https://www.suseso.cl/607/w3-article-496928.html .

[B34] Taylor D. M. O’Toole K. S. Ryan C. M. (2003). Experienced scuba divers in Australia and the United States suffer considerable injury and morbidity. Wilderness and Environmental Medicine 14 (2), 83–88. 10.1580/1080-6032(2003)014[0083:esdiaa]2.0.co;2 12825881

[B35] Thijssen D. H. Bruno R. M. van Mil A. C. Holder S. M. Faita F. Greyling A. (2019). Expert consensus and evidence-based recommendations for the assessment of flow-mediated dilation in humans. Eur. Heart J. 40 (30), 2534–2547. 10.1093/eurheartj/ehz350 31211361

[B36] Tremblay J. C. Grewal A. S. Pyke K. E. (2019). Examining the acute effects of retrograde *versus* low mean shear rate on flow-mediated dilation. J. Appl. Physiology 126 (5), 1335–1342. 10.1152/japplphysiol.01065.2018 30844335 PMC6589808

[B37] Turner R. T. (2000). Invited review: what do we know about the effects of spaceflight on bone? J. Applied Physiology 89 (2), 840–847. 10.1152/jappl.2000.89.2.840 10926671

[B38] Vakulenko D. Vakulenko L. (2023). “Evaluation of the adaptive capacity of the cardiovascular system during the Ruffier test determined by the results of morphological analysis of arterial pulsations recorded during blood pressure measurement using the Oranta-AO information system,” in Arterial oscillography: New capabilities of the blood pressure monitor with the Oranta-AO information system. Editors Liudmyla V. Natalia K. , 635, 307–324.

[B39] Vakulenko D. Vakulenko L. Barladin O. Khrabra S. Kadobnyj T. (2023). “Evaluation of transient states during the Martinet-Kushelevsky test, determined by arterial pulsations during blood pressure measurement in the Oranta-AO information system,” in Arterial oscillography: New capabilities of the blood pressure monitor with the Oranta-AO information system. Editors Vakulenko D. V. Vakulenko L. O. , 634.

[B40] Véliz A. Pereira R. Dörner A. Álvarez C. (2025). Bone mineral content determined by energy X-ray absorptiometry correlates with handgrip strength in Latin American divers. Front. Public Health, 13–2025. 10.3389/fpubh.2025.1591242 40726948 PMC12301340

[B41] Vogel T. Leprêtre P. M. Brechat P. H. Lonsdorfer‐Wolf E. Kaltenbach G. Lonsdorfer J. (2013). Effect of a short‐term intermittent exercise‐training programme on the pulse wave velocity and arterial pressure: a prospective study among 71 healthy older subjects. Int. Journal Clinical Practice 67 (5), 420–426. 10.1111/ijcp.12021 23574102

[B42] Weeldreyer N. R. De Guzman J. C. Paterson C. Allen J. D. Gaesser G. A. Angadi S. S. (2025). Cardiorespiratory fitness, body mass index and mortality: a systematic review and meta-analysis. Br. J. Sports Med. 59 (5), 339–346. 10.1136/bjsports-2024-108748 39537313 PMC11874340

[B43] WHO (2009). Global physical activity questionnaire: GPAQ version 2.0. Geneva, Switzerland: World Health Organization.

[B44] WHO (2021). WHO guidelines on physical activity and sedentary behaviour. Geneva. 33369898

[B45] Zamodics M. Babity M. Mihok A. Bognar C. Bucsko-Varga A. Kulcsar P. (2025). Evaluation of treadmill cardiopulmonary exercise testing and field measurement results in women's youth and adult national team water polo players. Heliyon 11 (1), e41131. 10.1016/j.heliyon.2024.e41131 39802000 PMC11719302

